# *Ab Initio* Density Functional Calculations and Infra-Red Study of CO Interaction with Pd Atoms on θ-Al_2_O_3_ (010) Surface

**DOI:** 10.1038/s41598-017-06405-7

**Published:** 2017-07-24

**Authors:** Chaitanya K. Narula, Lawrence F. Allard, Zili Wu

**Affiliations:** 1 Materials Science & Technology Division, Oak Ridge National Laboratory, Oak Ridge, TN 37831-6133 USA; 2 Chemical Science Division and Center for Nanophase Materials Sciences, Oak Ridge National Laboratory, Oak Ridge, TN USA

## Abstract

The *ab initio* density functional theoretical studies show that energetics favor CO oxidation on single Pd atoms supported on θ-alumina. The diffuse reflectance infra-red spectroscopy (DRIFTS) results show that carbonates are formed as intermediates when single supported Pd atoms are exposed to a gaseous mixture of CO + O_2_. The rapid agglomeration of Pd atoms under CO oxidation conditions even at 6 °C leads to the presence of Pd particles along with single atoms during CO oxidation experiments. Thus, the observed CO oxidation has contributions from both single Pd atoms and Pd particles.

## Introduction

The smallest metal “particles” are single supported metal atoms (SACs) which are also present in fresh catalysts and participate in catalytic reactions^1–4^. Among SACs, the single supported palladium appears to be unique in terms of its reactivity for gas phase reactions and organic reactions. For example, Abbet *et al*. showed that single Pd atoms supported on an MgO surface are catalytically active for CO oxidation^5^. Datye *et al*. found that single supported Pd atoms on alumina can oxidize CO even at room temperature^6^. Anderson *et al*., on the other hand, showed that single Pd atom on titania cannot oxidize CO^7^. We find that Pd atoms on alumina are inactive for NO oxidation^8^. Regarding organic reactions, the atomically dispersed Pd(II) sites exhibited exceptional performance for selective oxidation of crotyl alcohol to cinnamaldehyde^9^. Lee *et al*. showed that the hydrogenation of benzaldehyde over Pd1/TiO_2_ can be accomplished at room temperature while Pd/C is an ineffective catalyst^10^. 1,3-butadienes can be selectively hydrogenated to butenes over Pd1/graphene under mild conditions^11^. The hydrogenation of acetylene on over Pd1/Cu is possible at low conversion (10–20%) and moderate ethylene selectivity (30%)^12^. The highly selective and efficient 1-hexyne conversion to 1-hexene is possible over [Pd]mpg-C3N4^13^. The hydrogenation of succinic acid to γ-butyrolactone over atomically dispersed Pd catalyst has been reported but there is some uncertainty on the activity of single atoms since the catalyst is a mix of single atoms and nanoparticles^14^. In contrast, magnetite supported palladium single atoms have been shown to be ineffective for alkene hydrogenation^15^. Thus Pd SACs have high activity for oxidation and hydrogenation type reactions although there are some exceptions.

We have recently shown that inert substrate supported single Pt-atoms^16, 17^ are catalytically active for CO and NO oxidation. In contrast, we found that single supported Pd atoms are completely ineffective NO oxidation catalysts^8^. In this manuscript, the θ-Al_2_O_3_ supported single Pd atom and Pd particles are named Pd_s_/θ-Al_2_O_3_ and Pd/θ-Al_2_O_3_, respectively. Intrigued by reports from Anderson *et al*.^7^ showing lack of activity and Datye *et al*.^6^ describing room temperature CO oxidation combined with our results on lack of NO oxidation over Pd SACs^8^, we carried out *ab initio* density functional theoretical studies to gain insights into CO interaction with single supported Pd atoms. We find that energetics of CO oxidation on Pd single atoms are quite favorable regardless of Pd structure (cation or adatom) or alumina involvement. In order to find support for the proposed pathway, we carried out infra-red studies of CO oxidation reactions which show that carbonate intermediates form during CO oxidation suggesting that our proposed pathway for CO oxidation on Pd adatom is the most likely pathway. However, we also noticed absorptions assignable to bridging CO at 6 °C which suggests that Pd agglomeration begins even at low temperatures prompting us to repeat CO oxidation experiments (described in supplementary materials). Unfortunately, our attempts to repeat CO-TPR, reported by Anderson *et al*.^7^, were not successful since we did not have access to a CO-TPR system that could be cooled to 130 K, and the Pd-SACs agglomerated as soon as they were exposed to CO at room temperature. It is important to point out that Datye *et al*. also observed agglomeration in their operando X-ray absorption spectroscopic study at ~90 °C which was not present in their Pd single atoms dispersed on La_2_O_3_-γ-Al_2_O_3_ substrate. Our experiments show that rapid agglomeration of Pd atoms occurs even at low temperatures under CO oxidation conditions. This suggests that the observed CO oxidation has a contribution from agglomerated Pd also.

## Results and Discussion

### Proposed pathway for CO oxidation

Density functional theoretical studies for CO oxidation on single atom catalysts in the literature propose two structures for SACs (cation or adatom) and two pathways for CO oxidations^1–4^. The CO oxidation on cationic SACs generally proceeds with CO adsorption on single atoms which react with oxygen from substrate to form CO_2_ which is then eliminated. The substrate re-oxidizes by capturing oxygen from the reactant stream. The CO oxidation over adatom SACs proceeds with CO and oxygen adsorption on single atoms, their rearrangement to O-O-C=O or carbonate, and release of CO_2_. The substrate either is not involved in CO oxidation (e.g. alumina)^16, 17^ or participates^5^ by bonding with oxygen (e.g. MgO(FC)-Pd(CO)_2_O_2_) which is also bonded to single atoms. For CO oxidation on single Pd atoms supported on γ-alumina, the cationic structure has been proposed and CO oxidation has been proposed to proceed via CO adsorption on Pd atoms which reacts with oxygen from γ-alumina to form CO_2_^6^.

**Figure 1:**
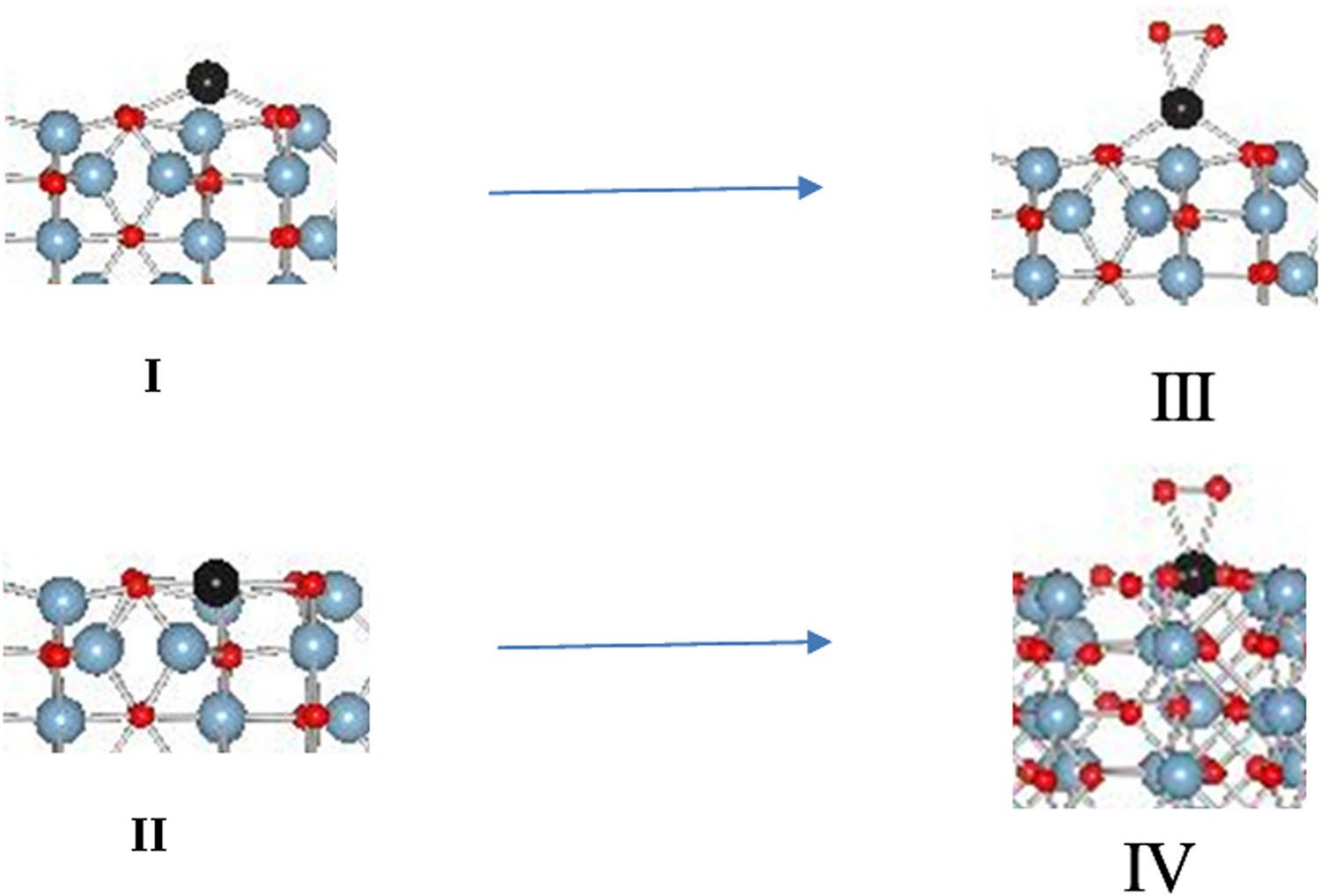
(**I)**. Pd adatom and (**II)**. Pd cation on θ-alumina (010) surface and corresponding oxidized species, (**III)** and (**IV)**.

Our recent work^8^ on NO interaction with Pd atoms supported on θ-alumina shows that Pd adatom, I, is more likely to be formed than Pd cation, II, because the Pd cation is a d^9^ species that is rare in Pd chemistry [Fig. 1]. Furthermore, the oxidized Pd adatom is more likely to represent Pd SACs since oxidized Pd adatom is tetra-coordinate^8^ which matches well with the reported EXAFS data^6^. Oxidized Pd cation, IV, is a six-coordinate configuration and does not match with the reported EXAFS data.

**Figure 2:**
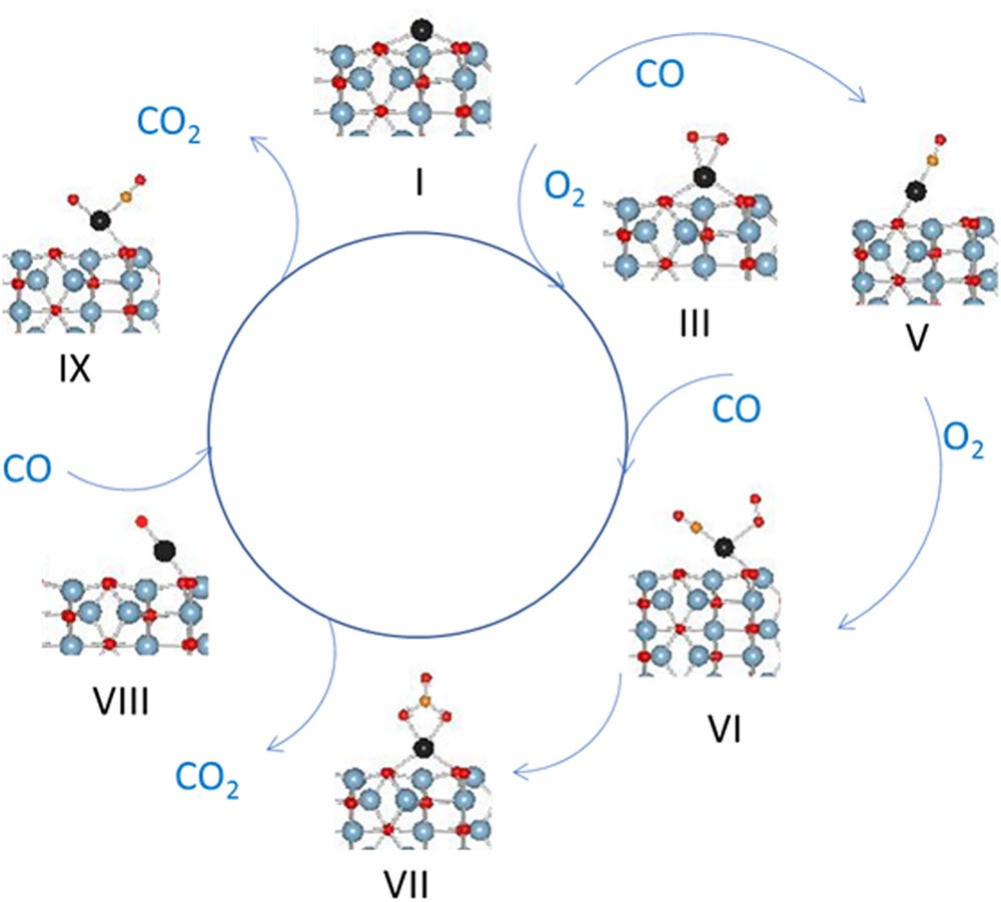
CO oxidation cycle on a single supported Pd adatom.

Here, we present our results on the CO oxidation pathway on an oxidized Pd adatom, III, which is more likely to represent Pd SAC under ambient conditions. The CO oxidation pathway on cationic Pd, II, is presented in Supplementary materials and the results clearly show that CO oxidation is quite facile in cationic Pd also. The rationale for employing θ-alumina instead of γ-alumina in preparation of model catalyst has been presented previously^16, 18^. The proposed pathway for CO oxidation on a Pd adatom proceeds via the scheme shown in Fig. 2. The total energy, Pd bond distances to surface oxygen, and magnetization for all intermediate configurations is summarized in Table 1. The oxidized configuration III reacts with CO via an exothermic reaction (−1.53 eV) to form configuration VI. The η2 oxygen of configuration III changes to a mono-dentate terminal oxygen in configuration VI. In addition, one of the two Pd-O bonds with surface oxygen breaks to accommodate CO. There is no magnetization associated with configuration VI, and PDOS analysis shows no vacant d-orbitals suggesting a d^10^ Pd oxidation state [Figure S1].

**Table 1:** Bonding Parameters and Magnetization values of configurations in Fig. 1 ^†^.

*Configuration*	*ΔE*_*I*_	*Total Energy (eV)*	*Pd-O bonds (Å)*		*Magnetic Moment*			
			*O1*	*O2*	*μ*_*Total*_	*μ*_*Mtotal*_	*μ*_*Ototal*_	
							O1	O2
I		−1315.889	2.22	2.20	0.0	—	—	—
III	−10.293	−1326.182	2.27	2.24	0.0	—	—	—
V	−16.805	−1332.694	—	2.08	0.0	—	—	—
VI	−26.595	−1342.484	—	2.12	2.0	0.22	0.0	0.0
VII	−29.640	−1345.529	2.25	2.18	0.0	—	—	—
ii	−26.869	−1342.758	2.41	2.18	0.0	—	—	—
VIII	−4.621	−1320.510	2.05	—	2.0	0.78	0.04	—
IX	−20.141	−1336.030	—	2.32	0.0	—	—	—

^†^Pd is bonded to surface oxygen O1 and O2. Magnetization spread over other surface atoms is not listed in the table. The data for configurations I are from ref. 18 and III and VIII are from ref. 8.

The carbonate formation (configuration VII) from configuration VI takes place via an exothermic process (−3.57 eV). The carbonate in configuration VII is a bi-dentate ligand and can be considered a 16-electron Pd species. There is no magnetization associated with configuration VII and PDOS does not show vacant d-orbitals, which supports a d^10^ oxidation state for Pd [Figure S1]. The bidentate bonding of carbonate to Pd is known for organopalladium complexes with a Pd-O bond of 2.06 Å and O-C-O angle of 113.5° ^19^. Our optimized Pd-O bond distances are 2.01 Å and O-C-O bond angle is 109.5°.

**Figure 3:**
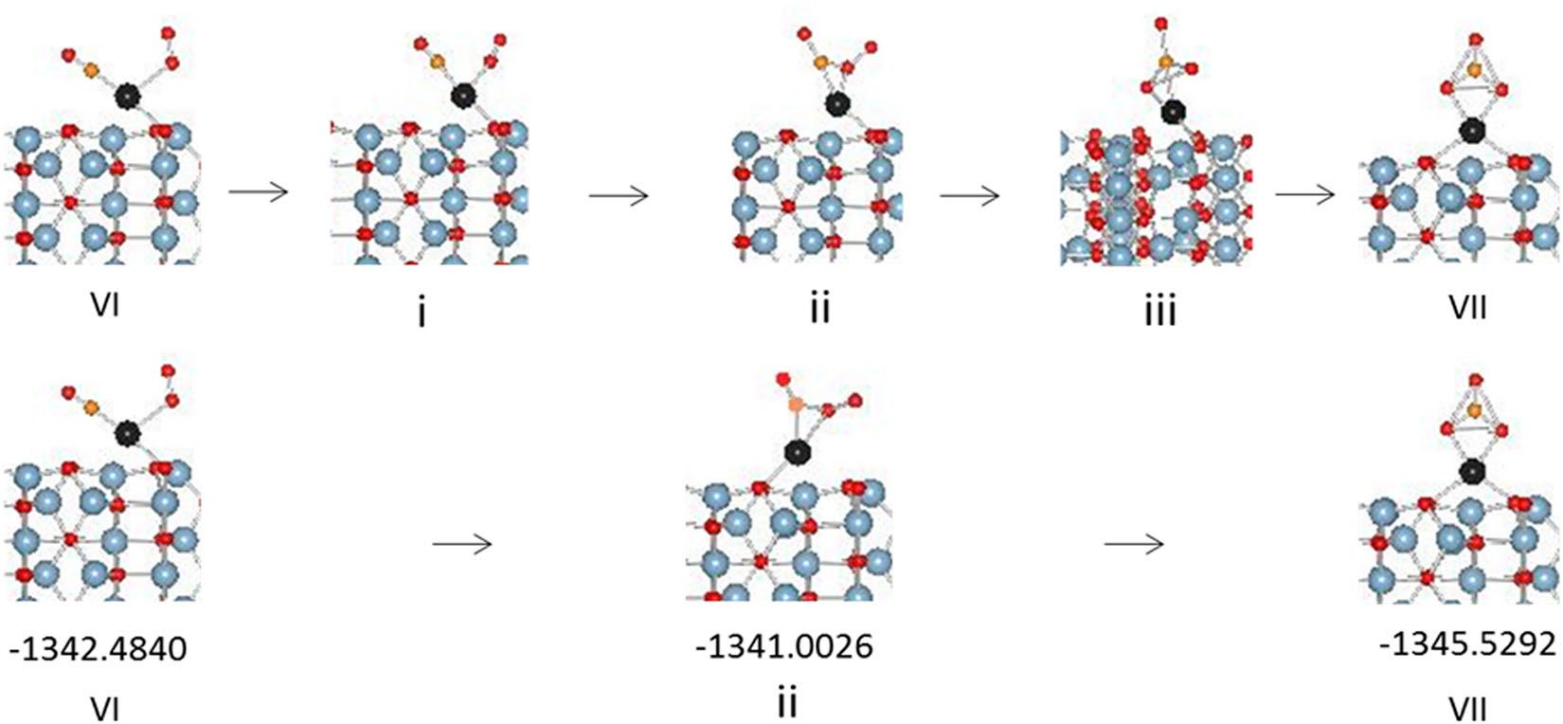
Transition states in carbonate formation over Pd adatom supported on θ-alumina (010) surface.

The three calculated transition states (i–iii) as well as optimized image ii are shown in Fig. 3. The image i optimized to a configuration that is very close to configuration VI in terms of structure and total energy while image iii optimized to a configuration close to configuration VII. The transformation of VI to transition state ii is an endothermic step (1.48 eV) which forms configuration VII via an exothermic step (−4.045 eV).

The loss of CO_2_ from VII is an endothermic process (2.06 eV) resulting in configuration VII which reacts with CO via an exothermic process (−0.73 eV) to form configuration VIII. The Pd in configuration VIII is in the d^10^ oxidation state with no magnetization associated with it. The loss of CO_2_ via an exothermic process (−2.82 eV) results in the formation of single Pd atom species I. Once the transient species I is formed, it has preference for reaction with CO over oxygen since its reaction with CO to form configuration V is more exothermic than that with molecular oxygen (−2.03 vs −1.51 eV). Configuration V is a d^10^ species with no magnetization associated with it and no empty d orbitals (Figure S1). Configuration V can react with molecular oxygen via an exothermic reaction (−1.00 eV) to form configuration VI.

**Figure 4:**
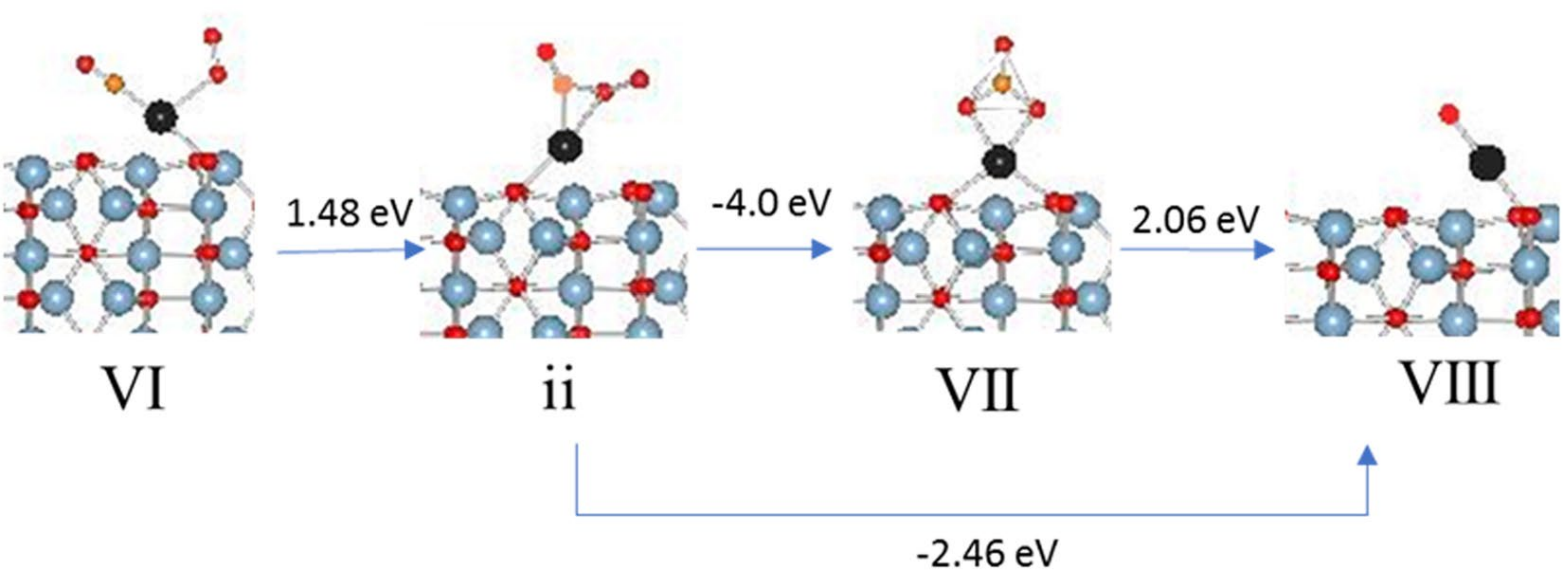
Energetics of CO_2_ release.

Another important point to consider is the O=C-O-O type species which has been proposed as an intermediate in CO oxidation over Pt nanoparticles^20^. Since there is only one Pd atom, the O=C-O-O species in configuration ii bonds to Pd via carbon and terminal peroxo oxygen [Fig. 4]. The lack of magnetization on Pd and the filled d-orbitals in PDOS suggest a d^10^ state for Pd. [Figure S1]. The formation ii from configuration VI is and endothermic event but the release of CO_2_ is an exothermic one. However, the rearrangement of ii to carbonate, VII, is highly exothermic event and is preferred over CO_2_ release.

The energetics of reactions in Fig. 1 are summarized as follows:

$$\begin{array}{lllll}{}^{\ast }{\rm{P}}{\rm{d}}\,({\rm{I}}) & {+{\rm{O}}}_{{\rm{2}}} & = & {}^{\ast }{\rm{P}}{{\rm{dO}}}_{{\rm{2}}}\,({\rm{III}}) & -{\rm{1.51}}\,\,{\rm{eV}}\\ {}^{\ast }{\rm{P}}{\rm{d}}\,({\rm{I}}) & +\mathrm{CO} & = & {}^{\ast }{\rm{P}}{\rm{dCO}}\,({\rm{V}}) & -{\rm{2.03}}\,\,{\rm{eV}}\\ {}^{\ast }{\rm{P}}{{\rm{dO}}}_{{\rm{2}}}\,(\mathrm{III}) & -{\rm{O}} & = & {}^{\ast }{\rm{P}}{\rm{dO}}\,(\mathrm{VIII}) & {\rm{5.58}}\,\,{\rm{eV}}\\ {}^{\ast }{\rm{P}}{{\rm{dO}}}_{{\rm{2}}}\,(\mathrm{III}) & +\mathrm{CO} & = & {}^{\ast }{\rm{P}}{{\rm{d}}({\rm{O}}}_{2})\,(\mathrm{CO})\,(\mathrm{VI}) & -{\rm{1.526}}\,\,{\rm{eV}}\\ {}^{\ast }{\rm{P}}{\rm{d}}\,{({\rm{O}}}_{2})\,(\mathrm{CO})\,(\mathrm{VI}) &  & = & {}^{\ast }{\rm{P}}{{\rm{d}}(\mathrm{CO}}_{3})\,(\mathrm{VII})\, & -{\rm{3.57}}\,\,{\rm{eV}}\\ {}^{\ast }{\rm{P}}{\rm{d}}\,{(\mathrm{CO}}_{3})\,(\mathrm{VII}) & -{{\rm{CO}}}_{{\rm{2}}} & = & {}^{\ast }{\rm{P}}{\rm{dO}}\,(\mathrm{VIII}) & {\rm{2.06}}\,\,{\rm{eV}}\\ {}^{\ast }{\rm{P}}{\rm{dO}}\,(\mathrm{VIII}) & +\mathrm{CO} & = & {}^{\ast }{\rm{P}}{\rm{d}}({\rm{O}})\,(\mathrm{CO})\,(\mathrm{IX}) & -{\rm{0.73}}\,{\rm{eV}}\\ {}^{\ast }{\rm{P}}{\rm{d}}\,({\rm{O}})\,(\mathrm{CO})\,(\mathrm{IX}) & -{{\rm{CO}}}_{{\rm{2}}} & = & {}^{\ast }{\rm{P}}{\rm{d}}\,({\rm{I}}) & -{\rm{2.82}}\,\,{\rm{eV}}\end{array}$$

The pathway suggests that CO oxidation is feasible on Pd adatoms and the reaction can proceed either via O=C-O-O intermediate which form via endothermic reaction from rearrangement of VI and then releases CO_2_ via an exothermic reaction or via CO_3_ intermediate which forms from VI via a highly exothermic reaction and then releases CO_2_ via an endothermic reaction. The rearrangement of O=C-O-O to CO_3_ is also a highly exothermic event. Infra-red studies, described in the next section, provide additional insights into CO interaction with Pd atoms.

### *In situ* Diffuse Reflectance Studies

**Figure 5:**
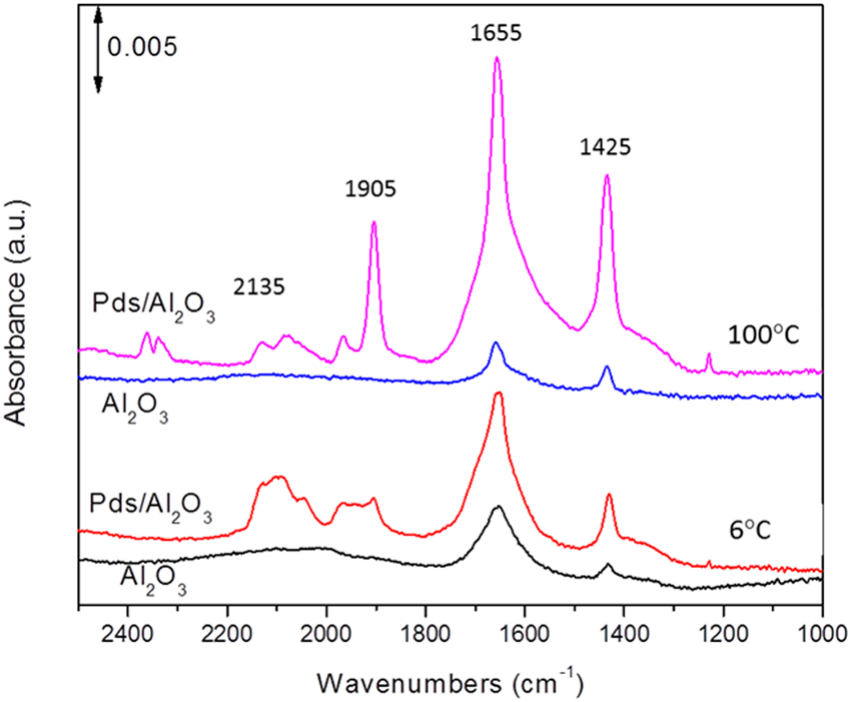
A comparison of *in situ* IR spectra during CO oxidation at 6 and 100 °C on θ-alumina and Pds/θ-alumina.

The catalyst sample Pds/θ-Al_2_O_3_ was cleaned under oxidizing conditions employing a mixture of 5% oxygen in helium. The results of CO adsorption studies on θ-alumina and Pds/θ-alumina in the presence of O_2_ at 6 and 100 °C are shown in Fig. 5. We selected 6 °C because Pd_s_/θ-Al_2_O_3_ has been shown to oxidize CO at room temperature and we expected negligible CO oxidation at this temperature.

Pure θ-Al_2_O_3_ does not exhibit any CO adsorption bands which are generally present in 2155–2245 cm^−1^ and 1050–1090 cm^−1^ range. The asymmetric and symmetric stretches of bicarbonate are seen at 1650 and 1438 cm^−1^, respectively. The Pd_s_/θ-Al_2_O_3_ sample shows bands in 1900–2150 cm^−1^ region typically associated with adsorbed CO. The bands at 2050, 2085, and 2135 cm^−1^ can be assigned to linear CO (a-top bound CO on Pd^0^ and Pd^2+^) while the bands at 1905 and 1970 cm^−1^ can be assigned to two-fold bridge CO^21^. This suggests that CO oxidation conditions induce Pd agglomeration even at 6 °C since single atoms cannot support bridged CO. The dominant peaks are carbonate peaks at 1655 and 1425 cm^−1^ which are assigned to bidentate carbonates in comparison with CO_3_ adsorption bands for organopalladium carbonates since single Pd atoms are isoelectronic with organopalladium carbonates. Several such compounds are known and the position of ʋ_s_C-O bands of carbonate has been reported to be in 1600–1650 cm^−1^ range and depending on the organo-species in organopalladium carbonates^20, 22–24^. Considering that the position of carbonate bands on θ-Al_2_O_3_ and Pd_s_/θ-Al_2_O_3_ is almost identical, the dramatic increase in intensity of carbonate bands on Pd_s_/θ-Al_2_O_3_ can be allocated to carbonates on Pd. It is important to note that previous detailed work on CO and CO_2_ interaction with Pd/γ-Al_2_O_3_ at 22 °C shows that carbonate and bicarbonate species are primarily due to interaction of CO_2_ with hydroxyls on alumina surface^25, 26^. No carbonates or bicarbonates were observed on the Pd particle surface. In a recent study of CO oxidation over alumina-supported platinum, Newton *et al*. conclude that the inability of θ-alumina to catalyze CO oxidation coupled with weak bidentate carbonate bands and missing adsorption in 1200–1300 cm^−1^ suggest that the species on θ-alumina are not a significant factor in CO oxidation reactions^27^.

**Figure 6:**
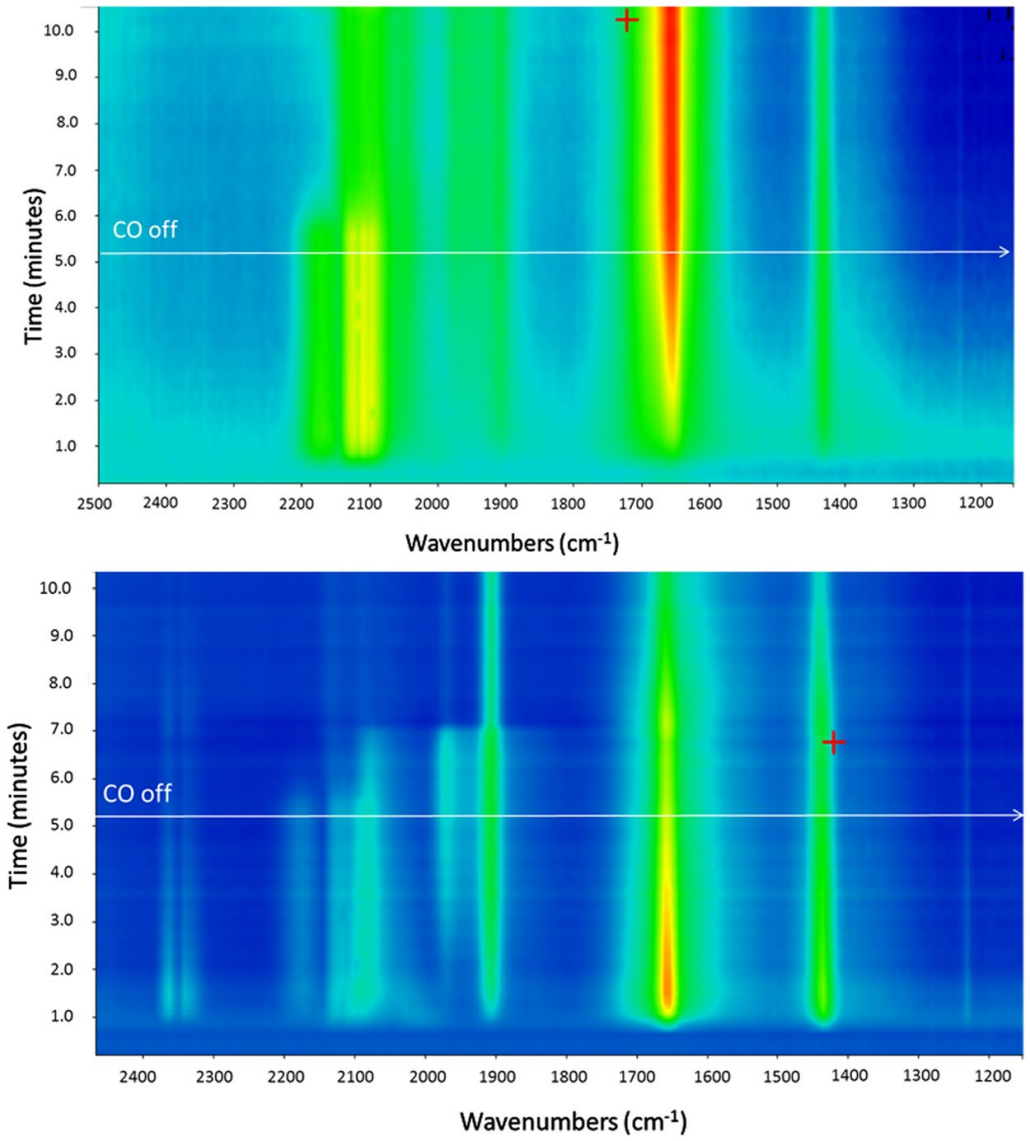
The evolution of IR bands during adsorption of CO + O_2_ for 5 minutes followed by desorption in O_2_ flow for 5 minutes at 6 °C (top) and 100 °C (bottom).

Increasing temperatures to 100 °C for CO oxidation results in several important changes in the IR of Pds/θ-alumina. First, two bands associated with physisorbed CO_2_ can be seen at 2040–2060 cm^−1^. Second, the carbonate bands at 1655 and 1425 cm^−1^ become very strong. Finally, there is a dramatic increase in 1905 cm^−1^ band for bridging CO bonded to Pd. The presence of physisorbed CO_2_ and increase in bidentate carbonate intermediate suggests that CO oxidation has accelerated at 100 °C which is commensurate with our experimental observations. The formation of Pd nanoclusters leads to increase in bridging CO bands. The IR of CO adsorption on θ-alumina sample does not change at 100°C and is comparable to the one observed at 6 °C suggesting that the carbonates formed on θ-alumina are stable at 100 °C. The time-resolved IR spectra during adsorption of CO + O_2_ for 5 minutes followed by O_2_ purge for 5 minutes at 6 °C are shown in Fig. 6 (top).

The fresh oxidized catalyst starts to adsorb CO in linear mode immediately with simultaneous appearance of carbonate bands. The bridging CO also starts to build up although it is quite week. At five minutes, the linear CO bands and carbonate bands are the strongest bands. Immediately after an O_2_ purge is started, the linear CO bands disappear within 30 seconds with concurrent strengthening of the carbonate bands. No physisorbed CO_2_ bands are observed in either CO or O_2_ pulse cycle. Increasing the temperature to 100 °C (bottom of Fig. 6) results in immediate appearance of physisorbed CO_2_ bands which continue to become weak during CO + O_2_ reaction and become very weak during the O_2_ purge. A similar trend is found for the carbonate IR peaks which are initially strong but start to decrease due to the loss of CO_2_ and continue to decrease in the O_2_ cycle. The linear CO peaks on single Pd atoms practically disappear as soon as the O_2_ cycle begins but the CO peaks on Pd nanoparticles take almost 2 minutes under O_2_ to become weaker. The bridging CO band increases in CO + O_2_ cycle and does not change after 2 minutes in the O_2_ cycle.

The observation of carbonate at 6 °C in a CO + O_2_ cycle which does not decrease in the O_2_ cycle, and lack of CO_2_ release supports our proposed mechanism which shows that the decomposition of carbonate is endothermic and the CO oxidation will stop after the catalyst surface is covered with carbonate species. At 100 °C, the release of CO_2_ is observed which is commensurate with our experimental observation that CO oxidation on Pds/θ-alumina begins just above 100 °C (Fig. 2). Our experiments also show that the agglomeration of Pd single atoms begins immediately under CO oxidation conditions at 6 °C but is very slow. This agglomeration accelerates at 100 °C resulting in particles that adsorb CO in the bridging mode.

### CO oxidation on single supported Pd atoms

In literature, the first report on CO oxidation catalyzed by isolated Pd atoms appeared in 2001. Abbet *et al*. showed that Pd atoms anchored on oxygen surface vacancies of MgO(100) thin films when exposed to oxygen followed by CO release CO_2_ with desorption at 260 and 500 K. Abbet el al. also showed that if the sequence is reversed i.e. if CO is adsorbed first before oxygen, CO_2_ formation is suppressed due to CO poisoning. Anderson *et al*., on the other hand, found no CO oxidation on Pd1/TiO_2_ in their TPR experiments in the –143 to 257 °C range, after treatment of Pd1/TiO_2_ samples with O_2_ at 127 °C and CO at −93 °C^7^. A definitive work from Datye *et al*. found that CO oxidation is quite facile on single Pd atoms. The kinetic studies show that the reaction order for single Pd is −0.2 for CO and +0.16 for O_2_ which changes to −1 for CO and +1 for O_2_ as single Pd atom agglomerate to Pd metal^6^. The strongest evidence for the activity of Pd comes from the operando XAS study of single Pd atoms supported on La_2_O_3_-γ-Al_2_O_3_ which clearly show that Pd-Pd bonds are not present in the fresh sample or after exposure to CO oxidation conditions in the temperature range of 40–90 °C.

Our theoretical studies show that oxidized Pd adatom is likely representative of Pd_s_/θ-Al_2_O_3_ and energetics favor CO oxidation. This pathway suggests that CO oxidation proceeds via carbonate (or O-O-C = O) intermediate. This is different from our previous finding that NO oxidation is not facile since there was no driving force to form O=N-O-O or nitrate intermediates^8^. The infra-red studies show that carbonate intermediates are indeed formed under CO oxidation conditions on Pd_s_/θ-Al_2_O_3_. However, we noticed the Pd atoms start to agglomerate at temperatures as low as 6 °C under CO oxidation conditions. This led us to repeat parts of the experiments, described by Anderson *et al*. and Datye *et al*., and the results are described in the Supplementary section. First, we attempted TPR experiment by flowing air over Pd_s_/θ-Al_2_O_3_ at 25 °C and then switched the gas to CO which resulted in instantaneous graying of the sample. After 30 minutes of CO flow, the reaction was stopped and sample examined by electron microscopy which showed that the sample contained primarily large Pd particles. Second, we carried out CO oxidation over Pd_s_/θ-Al_2_O_3_ and Pd/θ-Al_2_O_3_ and found both catalysts to be quite effective. Electron microscopy of samples after CO oxidation also showed extensive agglomeration of Pd atoms.

Thus, theoretical studies suggest that Pd_s_/θ-Al_2_O_3_ can be effective CO oxidation catalysts. But, the experimental work does not confirm the activity of Pd_s_/θ-Al_2_O_3_ because Pd agglomerates form as soon as the sample is exposed to CO oxidation conditions. In view of previous work by Datye *et al*. on CO oxidation activity of Pd atoms, we conclude that observed CO oxidation has contributions from both single atoms and particles formed from agglomeration of Pd atoms.

## Methods

The θ-Al_2_O_3_ supported single Pd atom and Pd particles are named Pd_s_/θ-Al_2_O_3_ and Pd/θ-Al_2_O_3_, respectively and their synthesis and characterization has been presented in a recent publication.

### Computational Method

The total energy calculations, based on ab initio DFT, were carried out employing the Vienna Ab Initio Simulation Package (VASP)^28^. A generalized gradient approximation (GGA) in the Perdew-Wang-91 form was employed for the electron exchange and correlation potential^29, 30^. The projector-augmented wave (PAW) approach for describing electronic core states was used to solve Kohn-Sham equations^31, 32^. The plane wave basis set was truncated at a kinetic energy cutoff of 500 eV. A Gaussian smearing function with a width of 0.05 eV was applied near Fermi levels. Ionic relaxations were considered converged when the forces on the ions were >0.03 eV/Å. We have previously described the details of construction of (010) alumina surface^18^. From bulk optimized θ-alumina, a 180 atom charge neutral 2 × 4 supercell was constructed^18^. The slabs were separated by a 15 Å vacuum to minimize spurious interaction by periodic images. A 4 × 1 × 4 Monkhorst-Pack mesh was used for surface calculations. Nudged elastic band method was employed to find transition states^33, 34^.

### Infrared Study during CO Adsorption

The detailed methods for CO oxidation by *in situ* diffuse reflectance Fourier transform infrared spectroscopy (DRIFTS) have been described previously^16^. In summary, a Nicolet Nexus 670 spectrometer fitted with a MCT detector cooled with liquid nitrogen was used for recording spectra. The system is equipped with an *in situ* chamber (HC-900, Pike Technologies) with a capability to heat samples to 900 °C. For CO oxidation study, all samples were first cleaned by heating to 150 °C under a flow of 25 mL/min 5% O_2_ in helium at a rate of 3 °C/min with a hold time of 1 h. The CO adsorption spectra were recorded after exposing the samples to 12.5 mL/min 2% CO/2% Ar/He plus 12.5 mL/min 5% O_2_/He for about 5 minutes and flushing the sample with 5% O_2_/He (25 mL/min) at 6 and 100 °C.

## Electronic supplementary material

Supplementary Materials
