# Supplementary material for: Ab Initio Density Functional Calculations and Infra-Red Study of CO Interaction with Pd Atoms on θ-Al2O3 (010) Surface
Source: Sci Rep. 2017 Jul 24;7:6231. doi: 10.1038/s41598-017-06405-7 (PMC5524828; doi:10.1038/s41598-017-06405-7)
Supplement: Supplementary file 1 — Supplementary Materials [file 41598_2017_6405_MOESM1_ESM.doc]

Supplementary Materials

**Ab Initio Density Functional Calculations and Infra-Red Study of CO Interaction with Pd Atoms on θ-Al_2_O_3_ (010) Surface**

*Chaitanya K. Narula,^1*^ Lawrence F. Allard,^1^ and Zili Wu^2^*

*^1^* Materials Science & Technology Division, Oak Ridge National Laboratory, Oak Ridge, TN, USA, 37831-6133

^2^Chemical Science Division and Center for Nanophase Materials Sciences, Oak Ridge National Laboratory, Oak Ridge, TN

E-mail: [narulack@ornl.gov](mailto:narulack@ornl.gov)

RECEIVED DATE

________________________

*This manuscript has been authored by UT-Battelle, LLC under Contract No. DE-AC05-00OR22725 with the U.S. Department of Energy. The United States Government retains and the publisher, by accepting the article for publication, acknowledges that the United States Government retains a non-exclusive, paid-up, irrevocable, world-wide license to publish or reproduce the published form of this manuscript, or allow others to do so, for United States Government purposes. The Department of Energy will provide public access to these results of federally sponsored research in accordance with the DOE Public Access Plan (*[*http://energy.gov/downloads/doe-public-access-plan*](http://energy.gov/downloads/doe-public-access-plan)*).*

***
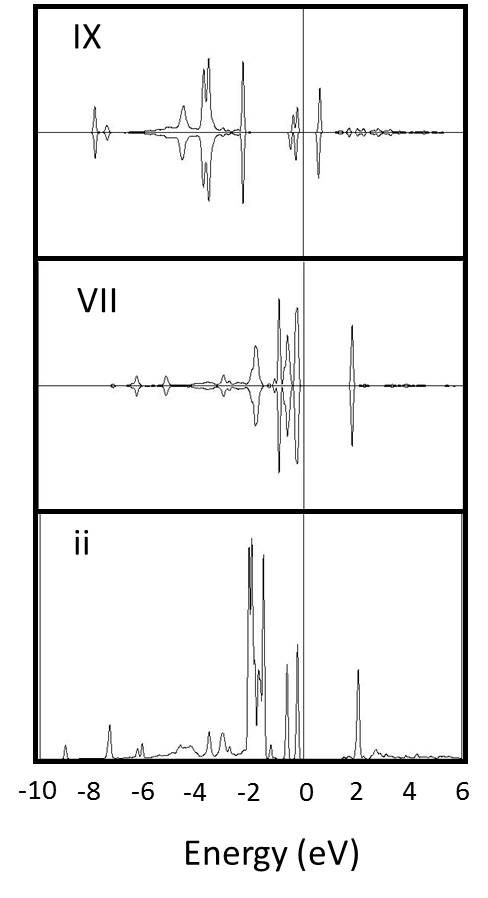
***

**
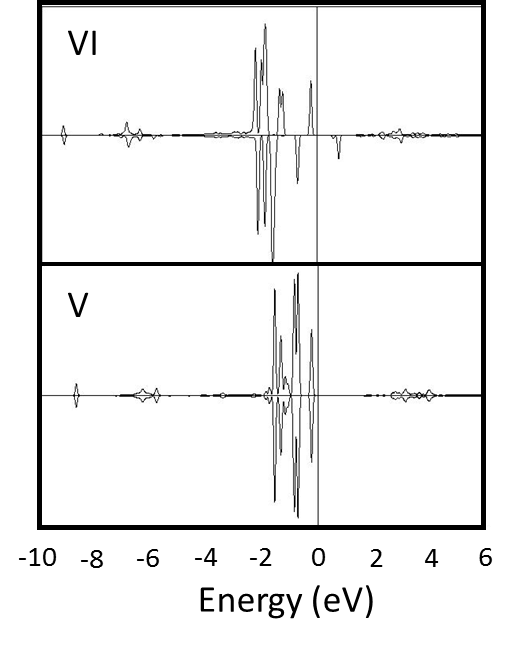
**

**Figure S 1**. PDOS of d-orbitals of configurations V-IX

**CO oxidation on cationic Pd.**

This pathway [Figure S2] is analogous to the pathway that was proposed by Datye et al. for Pd1/γ-alumina. The total energy, Pd bond distances to surface oxygen, and magnetizations for all intermediate configurations are summarized in Table S1.

**Table S1**. Bonding Parameters and Magnetization values of configurations in Figure S2†

| *Config.* | *ΔE_II_* | *Total Energy*  *(eV)* | *Pd-O bonds*  *(Å)*  *O1 O2 O3 O4* | | | | *Magnetic Moment* | | | | | |
| --- | --- | --- | --- | --- | --- | --- | --- | --- | --- | --- | --- | --- |
|  |  |  |  |  |  |  | *μ_Total_* | *μ_Mtotal_* | *μ_Ototal_* | | | |
|  |  |  |  |  |  |  |  |  | O1 | O2 | O3 | O4 |
| *S* I | -16.353 | -1323.319 | 2.06 | 2.41 | 2.15 | 1.96 | 0.68 | 0.12 | 0.0, 0.0,0.0, 0.34 | | | |
| *S* II | -16.219 | -1323.185 | 2.07 | 2.46 | 2.14 | 1.96 | 0.63 | 0.12 | 0.0, 0.0, 0.0, 0.32 | | | |
| *S* III | 6.54 | -1300.425 | - | 2.19 | 2.15 | - | 1.0 | 0.63 | -, 0.14, 0.16, - | | | |
| *S* IV | -4.165 | -1311.131 | 2.39 | 2.37 | 2.00 | - | 1.0 | 0.13 | 0.0, 0.00, 0.00, - | | | |
| *S* V | -20.194 | -1327.828 | 2.03 | 2.22 | - | - | 1.0 | 0.06 | 0.0, 0.0, -, - | | | |

† Pd is bonded to surface oxygen O1-O4. Magnetization spread over other surface atoms is not listed in the table. The data for configurations *S* III and *S* IV are from reference 8

The CO bonds to a Pd atom in configuration II via an exothermic reaction (-1.57 eV) to yield configuration *S* I. Configuration *S* I exhibits an elongated Pd-O bond and the magnetization spreads over Pd and surface oxygens. Partially filled dx2-y2 orbitals support a d^9^ Pd species in configuration *S* I [Figure S3]. Configuration *S* I changes to configuration *S* II via an endothermic step. The CO in configuration *S* II bends to bond with surface oxygen and the Pd remains in a d^9^ oxidation state. The loss of CO_2_ from configuration *S* II via an exothermic step results in configuration *S* III which adsorbs oxygen to form configuration *S* IV. Adsorption of CO by configuration *S* IV via an exothermic step results in configuration *S* V. The magnetization and empty dx2-y2 orbitals suggest a d^9^ oxidation state for Pd in configuration *S* V also [Figure S3]. The loss of CO_2_ from configuration *S* V regenerates configuration II.


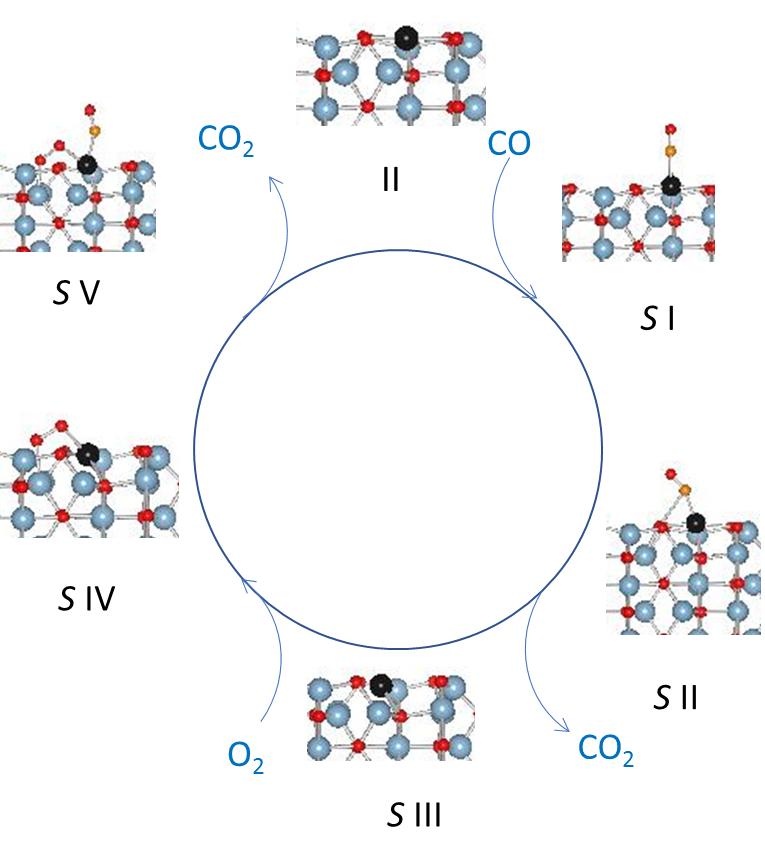


**Figure S2.** CO oxidation cycle on single supported Pd cation with alumina as an oxygen source

The energetics of reactions in Figure 12 are summarized as follows:

*Pd (II) + CO = *PdCO (*S* I) -1.57 eV

*PdCO (*S* I) = *PdCO (*S* II) 0.29 eV

*PdCO (*S* II) - CO_2_ = *Pd-O (*S* III) -0.35 eV

*Pd-O (*S* III) + O_2_ = *Pd-O(O_2_) (*S* IV) -1.92eV

*Pd-O(O_2_) (*S* IV) + CO = *Pd-O(O_2_)(CO) (*S* V) -0.98 eV

*Pd-O(O_2_)(CO) (*S* V) - CO_2_ = *Pd (II) -2.09 eV

Interestingly, this pathway has only one endothermic step - bending of Pd bonded CO to come in contact with surface oxygen for oxidation. In principle, CO oxidation can proceed via this pathway also.

**
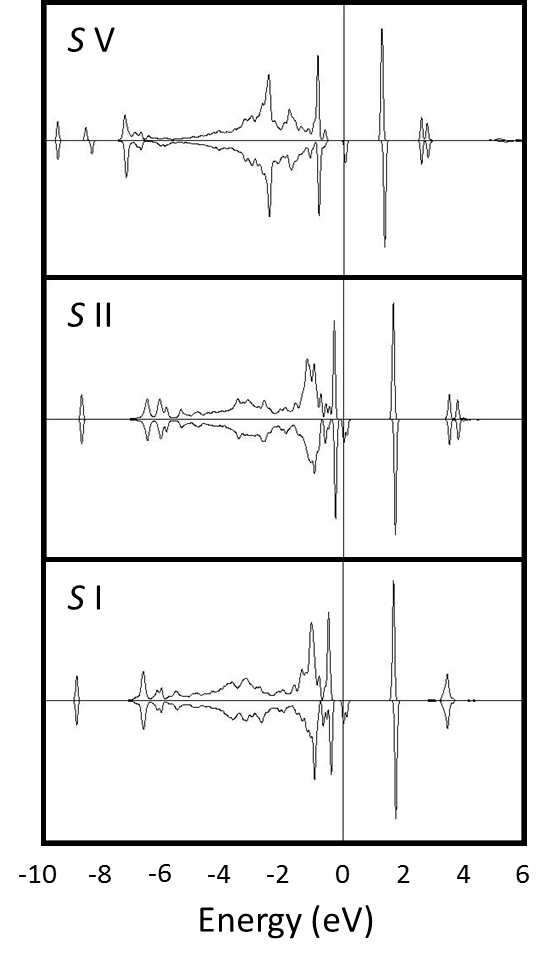
**

**Figure S3**. PDOS of d-orbitals of configurations S I, S II, and S V.

**EXPERIMENTAL**

The θ-Al_2_O_3_-supported single Pd atom and Pt particles are named Pd_s_/θ-Al_2_O_3_ and Pd/θ-Al_2_O_3_, respectively and their synthesis and characterization has been presented in a recent publication. The CO oxidation was carried out in a flow reactor and output gases were analyzed by MKS multi-gas infra-red analyzer. The powder catalyst (3.5 g) was loaded into the reactor between two quartz wool plugs. The GHSV was kept constant at 51.4k h^-1^ for all tests. The feed gas (total flow 3.0 litres/min) composition was 1% CO, 1% O_2_, and balance N_2_. The catalysts were heated under a flow of feed gas and the concentrations of gases were monitored at gas inlet temperatures in 100-200°C range. After the reactor reached a set temperature, the reactor was allowed to stay at that temperature for 5 minutes and gas concentration data were collected for 5 minutes and averaged. The residual CO was calculated by dividing CO concentration at set point with CO concentration in bypass mode.

The CO oxidation on Pd_s_/θ-Al_2_O_3_ initiates at 104°C gas inlet temperature [Figure S4] and is complete at 142°C. The overall light-off curve of CO oxidation over Pd_s_/θ-Al_2_O_3_ is shifted to a slightly higher temperature as compared with Pd/θ-Al_2_O_3_. Despite a significantly lower Pd loading in Pd_s_/θ-Al_2_O_3_, the light-off temperature is not dramatically different.

.

**Figure S4**. The CO oxidation light-off over Pd_s_-θ-alumina and Pd-θ-alumina

After a single cycle of CO oxidation, both Pd_s_/θ-Al_2_O_3_ and Pd/θ-Al_2_O_3_ samples turn dark. The high-angle annular dark-field (HAADF) STEM images of Pd_s_/θ-Al_2_O_3_ show large particles [Figure S5, right] with some single atoms. This suggests that CO oxidation conditions facilitate Pd sintering even at relatively low temperatures under CO oxidation conditions. The CO oxidation on single atoms, CO facilitated Pd sintering, and CO oxidation on sintered Pd particles must be taking place concurrently. For Pd/θ-Al_2_O_3_, the rafts become structurally undefined Pd particles (i.e large particles with no defined lattice structure) [Figure S5 right]. The CO oxidation over Pd/θ-Al_2_O_3_ initiates at 94°C and completes at 104°C. This difference can be assigned to a difference in palladium loading in Pd_s_/θ-Al_2_O_3_ and Pd/θ-Al_2_O_3_ or reaction pathways. Thus, Pds/θ-Al_2_O_3_ is effective CO oxidation catalyst although the Pd atoms rapidly sinter under CO oxidation conditions.


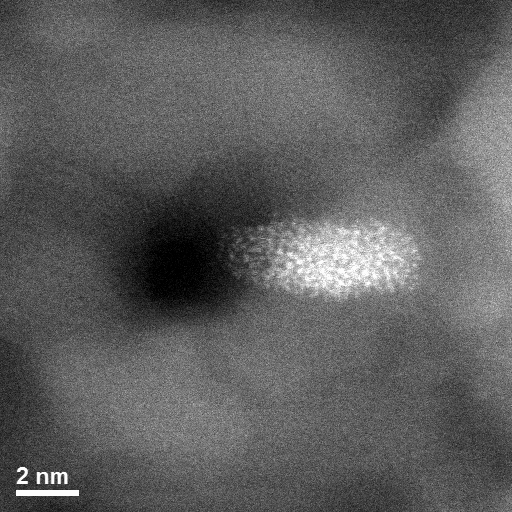


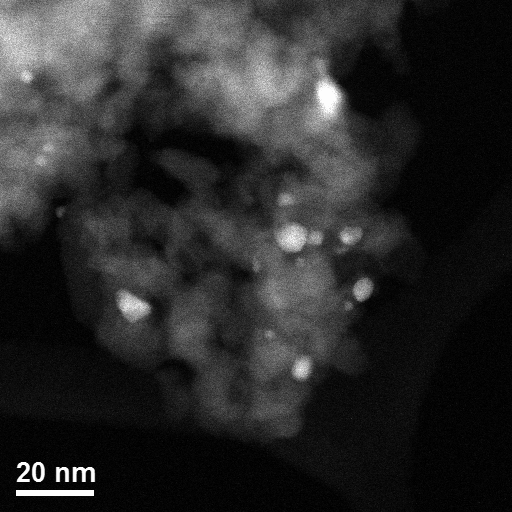

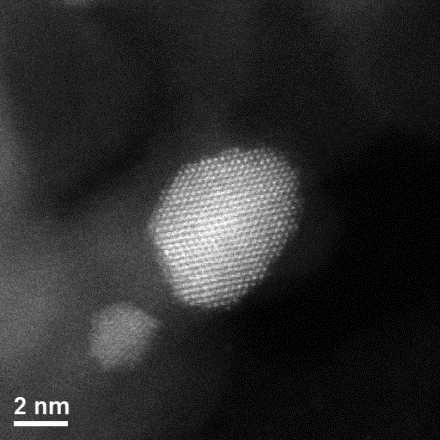

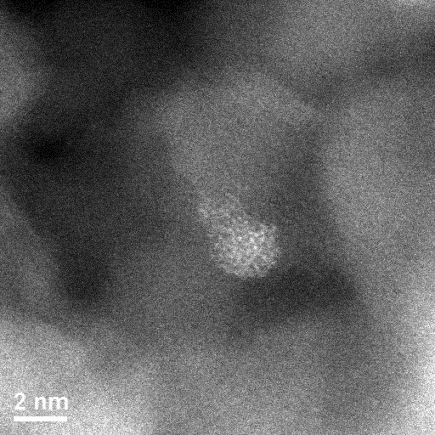


**Figure S5**. HAADF-STEM images of Pd_s_-θ-alumina (left) and Pd-θ-alumina (right) post CO oxidation experiment. The higher magnification images of inset area of Pd-θ-alumina are also shown.

The extensive sintering of Pd_s_/θ-Al_2_O_3_ during CO oxidation in our experiments led us to attempt CO-TPR experiment as described by Anderson et al. except that we planned CO adsorption at 25ºC. We were surprised to notice that the sample of Pd_s_/θ-Al_2_O_3_ turned gray as soon as it was exposed to CO. The HA-ADF images of Pd_s_/θ-Al_2_O_3_ sample after CO exposure of 30 minutes at 25ºC show that the sample is dominated by Pd nanoparticle of 5-10 nm [Figure S 6].


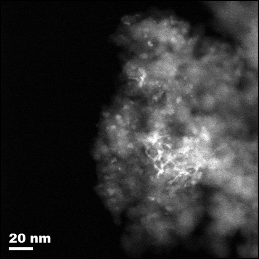

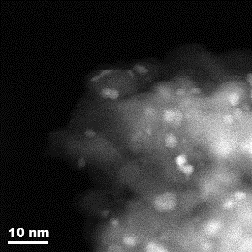


**Figure S6**. HAADF-STEM images of Pd_s_-θ-alumina post CO exposure at 25ºC.

These results show that CO oxidation conditions facilitate Pd sintering even at relatively low temperatures under a flow of CO and bulk of the single atoms agglomerate to Pd nanoparticles during CO oxidation.
